# The Invasive Mosquitoes of Canada: An Entomological, Medical, and Veterinary Review

**DOI:** 10.4269/ajtmh.21-0167

**Published:** 2022-07-11

**Authors:** Daniel A. H. Peach, Benjamin J. Matthews

**Affiliations:** Department of Zoology, University of British Columbia, Vancouver, BC, Canada

## Abstract

Several invasive mosquitoes have become established in Canada, including important pathogen vectors such as *Aedes albopictus*, *Ae. japonicus*, and *Culex pipiens*. Some species have been present for decades, while others are recent arrivals. Several species present new health concerns and may result in autochthonous seasonal outbreaks of pathogens, particularly in southern Canada, that were previously restricted to imported cases. This review provides an overview of current knowledge of the biological, medical, and veterinary perspectives of these invasive species and highlights the need for increased monitoring efforts and information sharing.

## INTRODUCTION

Mosquitoes (Diptera: Culicidae) are the world’s deadliest animals,^1^ vectoring myriad pathogens that result in untold pain and misery. Seventy nine native mosquito species are known from Canada,^2^^–^^4^ though several more are suspected to occur based on their distributions in the United States,^4^ and five invasive species are currently known.^2^^,^^4^^,^^5^ In this review, we define invasive mosquitoes as those that have been accidentally or deliberately introduced into areas beyond their native range and whose presence negatively impact the environment, the economy, or the society including human and animal health.^6^ Invasive mosquitoes threaten human and animal health as they can vector pathogens not previously known from Canada, or act as more efficient vectors of native pathogens. While the permanent establishment of many exotic mosquito-borne pathogens discussed here is unlikely in Canada, this may not always be the case due to novel mutations or climate change. Furthermore, localized seasonal outbreaks resulting from travel-related or other imported cases have occurred elsewhere.^7^ With increasing globalization and the landscape of emerging pathogens constantly changing, and as demonstrated by literature for other areas,^8^ an entomological, medical, and veterinary knowledge of the invasive mosquitoes of Canada is more important than ever.

## IMPORTANT MOSQUITO-BORNE PATHOGENS CURRENTLY OR FORMERLY ENDEMIC TO CANADA

Snowshoe hare virus (SSHV; Family: Bunyaviridae, genus: *Bunyavirus*) undergoes an enzootic transmission cycle in wild mammals with mosquitoes with non-*Culex* mosquitoes acting as the primary vectors.^9^ It is not clear what species are the principal hosts of SSHV, although small mammals are thought to be important in SSHV maintenance and amplification.^9^ Snowshoe hare virus is found across Canada^10^ as well as Alaska^11^ and parts of northern Eurasia.^12^ This virus has been reported to cause clinical encephalitis in humans,^13^ predominantly in children,^9^ and horses.^14^

Dog heartworm, *Dirofilaria immitis*, is a parasitic filarial worm that is an obligate parasite of mosquitoes and canids,^15^ although rare cases in other animals, such as humans, also occur.^16^ Endemic foci of *D. immitis* occur in many parts of Canada, particularly southern regions.^17^^,^^18^ A variety of mosquito species in the genera *Aedes*, *Culex,* and *Anopheles* vector *D. immitis*.^19^

West Nile virus (WNV; Family: Flaviviridae, genus: *Flavivirus*) undergoes an enzootic transmission cycle in avian hosts with mosquitoes in the genus *Culex* acting as primary vectors,^20^^,^^21^ though other mosquitoes with wide host ranges can carry WNV as well.^22^ The primary vectors of WNV in Canada are *Culex pipiens* and *Cx. restuans* in Eastern Canada, and *Cx. tarsalis* in Western Canada.^20^^–^^25^ *Culex* spp. are often ornithophilic, with some species feeding on humans as well. Ornithophilic biting behavior in the spring, by early emerging *Culex* spp. (such as *Cx. restuans* in Ontario) may vector enzootic transmission within local or migratory bird populations.^26^ During late summer vector species such as *Cx. tarsalis* or *Cx. pipiens* may increase their biting of humans^27^ leading to the transmission of WNV from birds to humans. West Nile virus first arrived in North America in 1999,^28^ and was first detected in Canada during 2001 in Ontario.^29^ By 2009, it had spread all the way west to British Columbia.^25^ West Nile virus can cause disease in several animals, including mortality in horses and birds.^30^^,^^31^

St. Louis encephalitis virus (SLEV; Family: Flaviviridae, genus: *Flavivirus*) undergoes an enzootic transmission cycle among birds and is vectored mainly by mosquitoes of the genus *Culex*.^32^ It may undergo vertical transmission and persist in mosquitoes through winter.^32^^,^^33^ During the 1970s, Canada experienced epidemics of SLEV, with the virus reported in Saskatchewan, Manitoba, Ontario, and Quebec,^10^ although in the United States it has been reported from coast-to-coast but predominantly in the southern States.^32^ St. Louis encephalitis virus can cause disease in horses as well.^34^

Jamestown Canyon virus (JCV; Family: Peribunyaviridae, genus: *Orthobunyavirus*) is transmitted primarily among wild ungulates by non-*Culex* mosquitoes.^9^ One study in the eastern United States found more than 20 field-collected mosquito species tested positive for JCV, with *Anopheles punctipennis*, *Coquillettidia perturbans*, and several *Aedes* spp. incriminated as likely vectors.^35^ Jamestown Canyon virus is widespread in temperate North America and, while human infections and disease are rare, they are likely underrecognized.^36^

Cache Valley virus (CVV; Family: Peribunyaviridae, genus: *Orthobunyavirus*) is transmitted primarily among ungulates by non-*Culex* mosquitoes.^9^ In the Canadian prairies, CVV has been isolated from *Aedes vexans*, *Culiseta incidens*, and *Culex *tarsalis.^37^ In rare instances, CVV can cause disease in humans,^38^ and congenital malformations in sheep and goats.^39^^,^^40^

The human malaria parasite, *Plasmodium vivax*, is transmitted by mosquitoes of the genus *Anophleles*. *Plasmodium vivax* was formerly endemic to parts of Eastern Canada,^41^ and the southern interior of British Columbia.^42^ Contemporary imported cases continue to result in local malaria outbreaks.^43^

Eastern equine encephalitis virus (EEEV; Family: Togaviridae, genus: *Alphavirus*) undergoes an enzootic transmission cycle among passerine birds and mosquito vectors.^32^ Eastern equine encephalitis virus is vectored between birds by the mosquito *Culiseta melanura*,^44^ which does not bite humans.^45^ Transmission of EEEV to humans and other mammals occurs through mosquitoes that feed on passerine birds and mammals, including *Coquillettidia perturbans*, *Cs. morsitans*, *Culex* spp., and some mosquitoes of the genus *Aedes*.^32^^,^^46^ In Canada, EEEV is found in Ontario and Quebec and can cause mortality in humans and horses.^44^ Domestic poultry has been reported in some cases to suffer a decrease in egg production as a result of infection with EEEV,^47^ and even mortality.^47^

Western equine encephalitis virus (WEEV; Family: Togaviridae, genus: *Alphavirus*) is transmitted between birds and mammals by a variety of mosquitoes, although the western encephalitis mosquito, *Cx. tarsalis*, is thought to be the most important vector.^32^ Western equine encephalitis virus can cause mortality in humans and horses,^32^ and it may affect domestic poultry as well including decreased egg laying.^48^ In Canada, WEEV is found from British Columbia to the Great Lakes.^10^

## SELECT EXOTIC PATHOGENS RELEVANT TO THIS REVIEW

Dengue virus (DENV; Family: Flaviviridae, genus: *Flavivirus*) is an arbovirus ubiquitous in the tropics that is vectored by some mosquitoes in the genus Aedes,^49^ primarily between humans but also nonhuman primates.^50^ There are several serotypes of dengue individuals who experience a subsequent infection with a different serotype are at increased risk of developing severe dengue.^51^

Japanese encephalitis (JEV; Family: Flaviviridae, genus: *Flavivirus*) undergoes enzootic transmission between birds and pigs and has recently spread from southeast Asia into Australia.^52^ This virus is the leading cause of encephalitis in eastern and southern Asia and is primarily vectored by mosquitoes of the genus *Culex*, although some *Aedes spp.* also act as vectors.^52^^–^^54^

Usutu virus (USUV; Family: Flaviviridae, genus: *Flavivirus*) is primarily vectored by *Culex* mosquitoes, and some members of the genus *Aedes.* Usutu virus was previously only known from Africa, but it has recently spread to Europe.^55^ Usutu virus primarily circulates in humans and birds, where it can cause encephalitis in humans^56^ and has caused severe mortality in bird populations that have not developed immunity.^55^

Yellow fever virus (YFV; Family: Flaviviridae, genus: *Flavivirus*) is transmitted among humans and other primates primarily by some *Aedes*, *Sabethes*, and *Haemogogus* mosquitoes.^57^ There is a vaccine for YFV; however, it has historically been considered a very dangerous pathogen.^57^ Yellow fever virus is primarily tropical in distribution; however, sporadic outbreaks have occurred as far north as New York City and Philadelphia,^57^ and there is risk of travel-related cases initiating autochthonous transmission cycles.^58^

Zika virus (ZIKV; Family: Flaviviridae, genus: *Flavivirus*) is transmitted between nonhuman primates and humans, primarily by many mosquitoes of the genus *Aedes*, although there are other transmission routes.^59^ Travellers returning from areas with endemic ZIKV may be at risk of initiating autochthonous transmission if competent vectors are present.^59^

Chikungunya (CHIKV; Family: Togaviridae, genus: *Alphavirus*) is primarily vectored by some *Aedes* mosquitoes.^60^ Nonhuman primates serve as potential reservoir or amplifying hosts,^60^ although vertical transmission in mosquitoes has also been reported.^61^

La Crosse virus (LACV; Family: Peribunyaviridae, genus: *Orthobunyavirus*) is the primary cause of viral encephalitis in children in the United States.^9^ The primary LACV vectors, *Aedes triseriatus* and *Ae. albopictus*, have a limited distribution within Canada.^4^^,^^5^

Rift Valley fever (RVFV; Family: Phenuiviridae, genus: *Phlebovirus*) is known from Africa and Arabia where it causes morbidity and mortality in humans and ruminants and is vectored by several pathways, including mosquitoes of a variety of genera.^62^

## INVASIVE MOSQUITOES KNOWN FROM CANADA

### *Aedes aegypti* distribution.

The yellow fever mosquito, *Aedes* (*Stegomyia*) *aegypti* (L.), originated in sub-Saharan Africa where its sylvatic form can still be found today.^63^ As it adapted to a synanthropic lifestyle, this species managed to spread to tropical and subtropical areas around the globe via human-assisted dispersal, particularly in association with ship traffic connected with the slave trade.^64^ One of the most globally widespread species in tropical and subtropical environments, *Ae. aegypti* has been present in North America for centuries but is intolerant of temperate winters.^65^ While *Ae. aegypti* has historically been limited to areas with mean January temperatures above 10°C,^65^ there are sporadic northern populations that exist in areas where mean January temperatures get as low as about 2°C.^66^ Habitat models for *Ae. aegypti* do not predict suitable year-round climate conditions for this species in Canada now or in the near future.^67^^,^^68^

**Figure 1.**
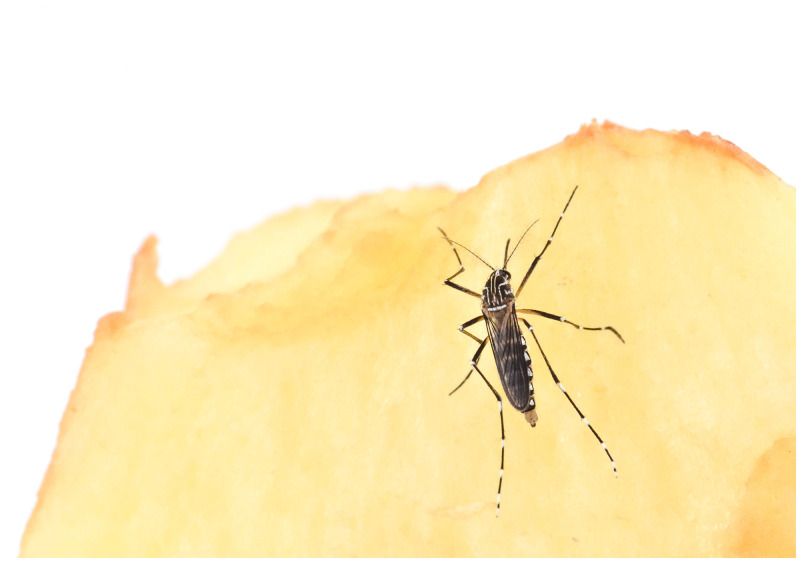
Female *Aedes aegypti*, photo by Adam Blake. This figure appears in color at www.ajtmh.org.

In 2016 and 2017, low numbers of *Ae. aegypti* were reported from Southern Ontario, representing the first records of this species in Canada^5^ (Figure 1). A record from southern Quebec in the summer of 2017 also exists.^69^^,^^70^ Although these records are believed to represent transient incursions,^5^ *Ae. aegypti* is thought to have managed to persist through the winter in other cooler locales as larvae in warm subterranean microenvironments.^66^

### Life history.

Historically, a sylvatic tree-hole breeder that fed on animals, almost all populations of *Ae. aegypti* now preferentially blood-feed on humans,^63^^,^^71^^–^^73^ are adapted to human-altered habitats, and breed in a wide variety of artificial containers such as tires, gutters, vases, and buckets^74^ as well as indoor and underground aquatic habitats.^75^ Eggs are deposited near the water surface, hatch when the water level rises, and are resistant to desiccation.^72^^,^^76^^,^^77^ They are aggressive biters that can feed on multiple hosts during a single gonotrophic cycle, increasing the risk of pathogen transmission.^78^ Adult female *Ae. aegypti* are primarily diurnal^79^^,^^80^ and readily enter human habitations to seek a blood meal or rest^72^; however, they are weak fliers and don’t often fly more than a few hundred meters from breeding sites unless inadvertently transported by humans.^81^

### Taxonomy and identification.

**Figure 2.**
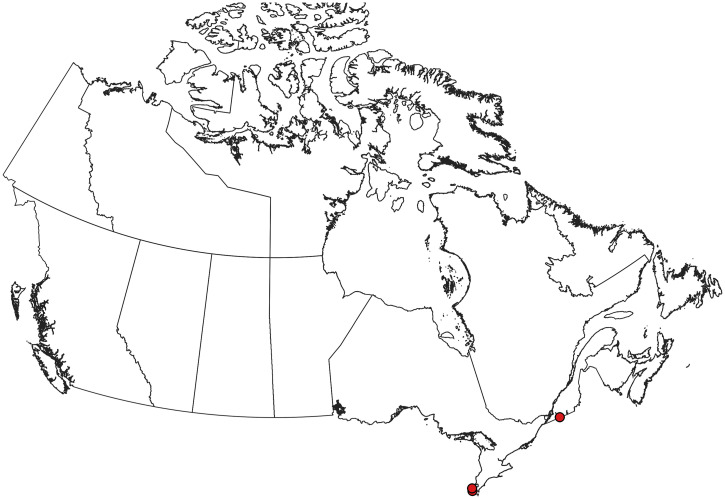
Collection records of *Aedes aegypti* in Canada.^5^^,^^70^ Note that these records are of ephemeral introductions that are not believed to represent established populations. This figure appears in color at www.ajtmh.org.

*Aedes aegypti* is a small black mosquito with stripes of white scales on the tarsomeres and a lyre-shaped pattern of white scales on the scutum^74^^,^^82^ (Figure 2). It looks similar to *Ae. sierrensis, Ae. albopictus*, *Ae. japonicus*, and *Orthopodomyia* spp.; however, the presence of white scales on both the base and apex of tarsomeres of *Ae. sierrensis*, prominent longitudinal middorsal white stripe of *Ae. albopictus*, the bronze-scaled lyre-shaped pattern on the scutum of *Ae. japonicus*, and the lack of distinct stripes of white scales on the fore tarsi of *Orthopodomyia* spp. can be used to separate these species from *Ae. aegypti*.

### Public health and veterinary importance.

*Aedes aegypti* is thus far only believed to present in Canada on a seasonal basis during the summer months.^5^ However, *Ae. aegypti* is a principal vector of several important arboviruses including DENV,^72^ YFV,^72^ ZIKV,^83^ and CHIKV.^84^ The CHIKV and ZIKV are among the most frequent travel-acquired pathogens in Canada,^9^^,^^85^^,^^86^ and the presence of *Ae. aegypti* in Canada, even on an ephemeral basis, raises concerns for autochthonous seasonal transmission of imported arboviruses.

### *Aedes albopictus* distribution.

The Asian tiger mosquito, *Aedes* (*Stegomyia*) *albopictus* (Skuse), is native to southeast Asia but has expanded is range to include an almost global occupation of tropical to temperate habitat.^67^ Its intercontinental dispersal is thought to be largely due to its use of used tires as breeding habitat,^87^^,^^88^ and within continents it is likely to spread via human-assisted means such as car travel.^89^

**Figure 3.**
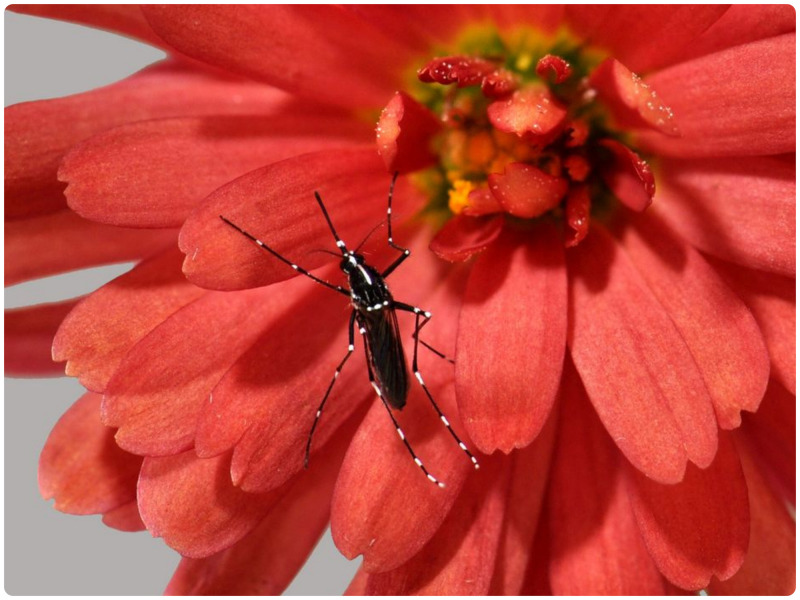
Female *Aedes albopictus*, photo by Ary Faraji. This figure appears in color at www.ajtmh.org.

*Aedes albopictus* was first detected breeding in the United States in 1985^90^ and it has now spread throughout much of the continental United States.^67^^,^^68^ This species was documented in Southern Ontario in 2002^2^ and was found to be established in 2019.^5^* Aedes albopictus* is currently only known in Canada from extreme Southern Ontario (Figure 3), but it has been intercepted in used tires in Seattle,^91^ not far from the British Columbia border and climate models predict it could establish in several other parts of southern Canada including Quebec, the Prairies, and the southern Maritimes.^68^ Due to the propensity of *Ae. albopictus* to spread via human-assisted dispersal and the concentration of the Canadian population in areas that contain suitable climate, or climate that is predicted to become suitable, for *Ae. albopictus*, this species may spread further within Canada. However, this spread may be slow, sporadic, or limited due to the climatic conditions that are currently marginal for its survival.

### Life history.

*Aedes albopictus* is an opportunistic daytime blood-feeder on a wide range of animals^74^^,^^92^ that is found in a variety of environments. *Aedes albopictus* is very flexible in its use of breeding habitat, using a wide variety of artificial containers and even natural habitats like tree holes.^74^ The larvae of *Ae. albopictus* are often able to outcompete the larvae of other species that share breeding habitats with them.^93^^,^^94^ At the northern limit of its range in the United States, *Ae. albopictus* is more abundant in areas with higher mean winter temperatures and March precipitation.^95^ Diapausing *Ae. albopictus* eggs that are cold-acclimated can survive at −10°C for up to 24 hours,^96^ surviving winters that reach lows of −10°C with apparent ease.^97^ How *Ae. albopictus* overwinters at the northern limits of its range are unknown, although it has been speculated that this mosquito may have adapted its life history strategy to overwinter as adults in human-made artificial habitats that provide warm microhabitat.^5^ The survival of eggs or larvae in similar human-made habitats is another possibility, as is a physiological adaptation. Research in this area would be useful in understanding the invasion ecology of this important species.

### Taxonomy and identification.

**Figure 4.**
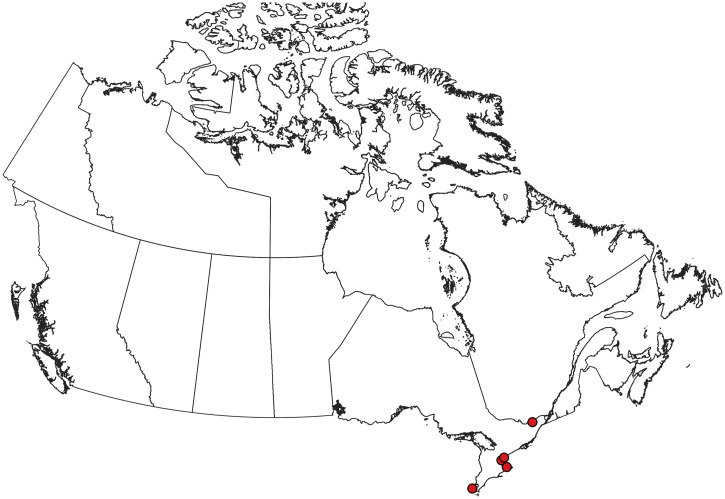
Collection records of *Aedes albopictus* in Canada.^5^ Only the population in Windsor, ON, is reported to be established. Other records are of ephemeral introductions that are not believed to represent established populations. This figure appears in color at www.ajtmh.org.

*Aedes albopictus* is a dark mosquito with bands of white scales on its tarsomeres, an entirely white-scaled 5th hind tarsomere, and a distinct longitudinal stripe of white middorsal scales on its scutum^74^ (Figure 4). *Aedes japonicus* and *Ae. aegypti* can be mistaken for *Ae. albopictus* due to similar patterns of silvery-white and black scales on the legs, but these species have patterns of scales on the scutum that differ from the middorsal white stripe of *Ae. albopictus*.

### Public health and veterinary importance.

*Aedes albopictus* is a very serious public health concern in much of the world due to its vector competency for exotic arboviruses like ZIKV,^98^ CHIKV,^99^ DENV,^100^ and YFV,^101^^,^^102^ as well as endemic arboviruses like WNV,^22^ EEEV,^103^^,^^104^ or emerging North American arboviral threats that may spread from the United States to Canada, such as LACV.^9^ Chikungunya and ZIKV are the common travel-acquired pathogens in Canada,^85^^,^^86^^,^^105^ and the establishment of *Ae. albopictus* may allow for imported cases to result in short-lived autochthonous arbovirus outbreaks, a pattern that occurs in other temperate locations^106^^–^^108^ and has occurred in Canada with other pathogens.^43^ *Aedes albopictus* is also a potential vector of dog heartworm.^19^

### *Aedes japonicus* distribution.

**Figure 5.**
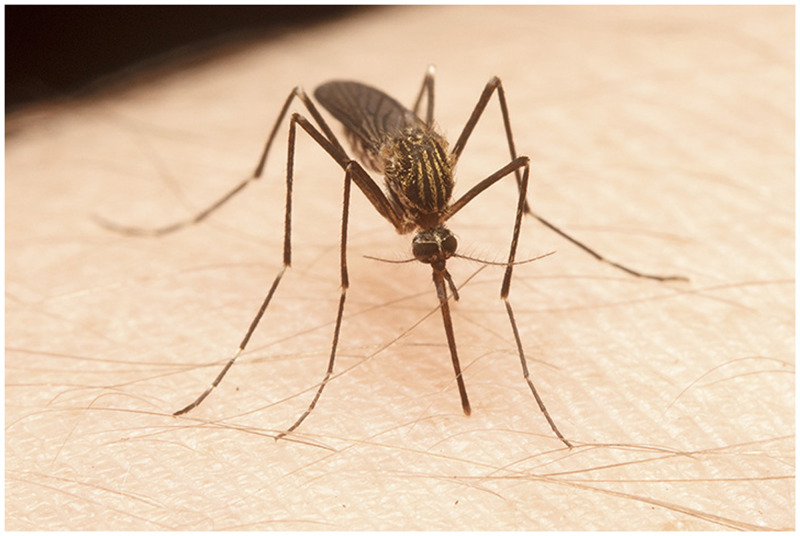
Female *Aedes japonicus*, photo by Sean McCann. This figure appears in color at www.ajtmh.org.

The Asian bush mosquito, *Aedes* (*Hulecoeteomyia*) *japonicus* (Theobald), is endemic to southeast Asia,^109^ but has expanded its global range drastically over the last several decades. There are four subspecies of *Ae. japonicus* (*Ae. j. japonicus*, *Ae. j. shintiensis*, *Ae. j. yaeyamensis*, and *Ae. j. amamiensis*)^109^ of which *Ae. j. japonicus* is the only one currently known from North America.^110^ Since its introduction to North America in 1998^111^ *Ae. j. japonicus* has spread over much of the continent and is projected to continue spreading.^112^ In Canada, it is now known from British Columbia^113^ including Vancouver Island,^114^ Ontario,^115^^,^^116^ Quebec,^117^^,^^118^ Newfoundland,^119^ New Brunswick,^120^ and Nova Scotia (J. Ogden, pers. comm) (Figure 5).

Genetic evidence indicates that *Ae. j. japonicus* arrived in eastern North America via at least two separate introductions from Europe,^121^ and similar studies suggest that populations in western North America are derived from these populations.^122^ Evidence from the United States suggests that human-assisted dispersal is the main mode of *Ae. j. japonicus* expansion within North America.^123^

Suitable habitat exists for *Ae. j. japonicus* on Prince Edward Island^112^ and it may only be a matter of time until this species arrives there. Climate change may also lead to suitable habitat for *Ae. japonicus* in Western Ontario and parts of the Prairies over the coming decades.^112^ Due to its propensity to spread,^124^ its mode of spread, the existence of unoccupied suitable habitat, and projections for an increase in suitable habitat over the coming decades, *Ae. j. japonicus* is likely to continue to spread within Canada.

### Life history.

*Aedes j. japonicus* breeds in tree holes, rock pools, and human containers such as used tires, bird baths, and discarded buckets,^110^ often containing decaying organic matter, and its distribution is correlated with forested or bushy areas.^125^ Adults are diurnal and crepuscular in their biting activity and take blood from mammals, including humans, and also from birds.^110^ *Aedes japonicus* is tolerant of cold temperatures, emerging earlier and active later in the season than other species that occupy similar ecological niches.^109^^,^^126^ *Aedes j. japonicus* can overwinter in the egg stage or the larval stage depending on climate and can produce multiple generations per year.^110^ Warm summer temperatures may prevent *Ae. j. japonicus* colonization in some areas.^127^ Laboratory studies have shown that eggs cannot survive extended exposure to temperatures lower than −9°C and that larvae cannot develop at temperatures greater than 31°C.

While there is evidence for competition between *Ae. j. japonicus* and other mosquitoes altering the assemblage of mosquito species in some areas,^127^ results from southern Ontario found no change in the species assemblage of mosquitoes with the arrival of *Ae. j. japonicus*,^116^ perhaps indicating such effects only occur under specific ecological contexts or at certain scales.

### Taxonomy and identification.

**Figure 6.**
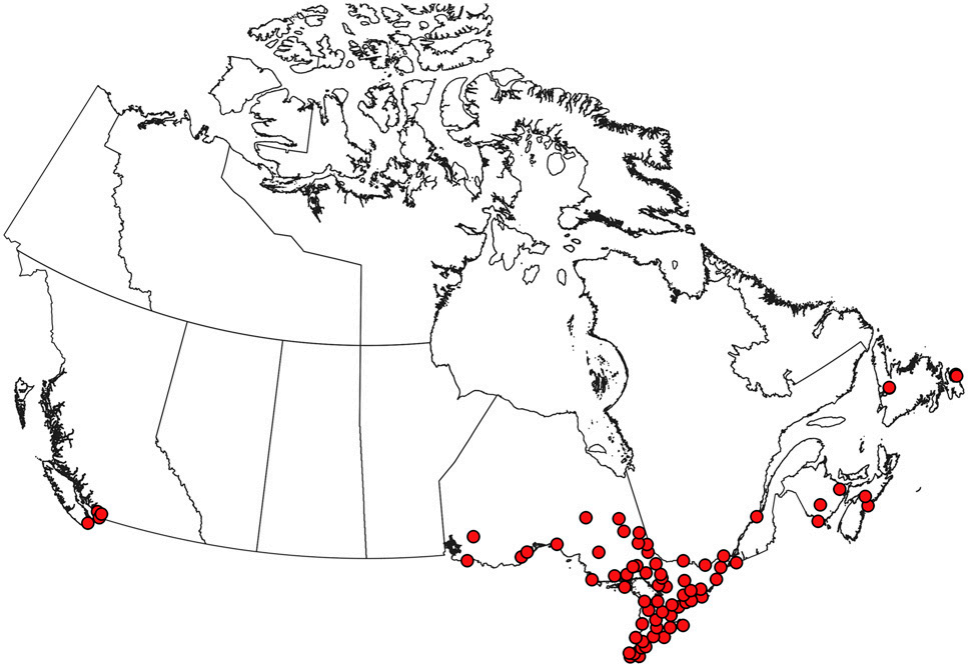
Collection records of *Aedes japonicus* in Canada.^114^^–^^120^^,^^219^^,^^220^ This species is established and widespread in southern Canada outside of drier areas. This figure appears in color at www.ajtmh.org.

A dark mosquito with bands of pale scales on the tarsomeres and a lyre-shaped pattern of bronze scales on the scutum (Figure 6). Adults of *Ae. j. japonicus* can be distinguished from other Canadian species by the lack of a basal band of pale scales on hind tarsomere 4, dark-scaled abdominal tergites, and a pedicel that usually has more pale scales than dark scales.^2^^,^^109^ A closely related species, *Ae. koreicus*, has established in Europe,^128^ outside of its native range in Asia, and maybe an invasive species of concern to Canada. *Aedes togoi* can be mistaken for *Ae. japonicus* due to similar patterns of scales on the legs and scutum, but the bands of pale scales on the tarsomeres of *Ae. japonicus* are present only at the base of each tarsomere.

#### Public health and veterinary importance.

*Aedes japonicus* is an aggressive biter that has been implicated as a vector of JEV in its native range,^54^ and may be a competent vector of WNV,^22^ EEEV,^104^ SLEV,^129^ RVFV,^130^ DENV and CHIKV,^131^ and CVV.^132^ This species is believed to be a significant vector of LACV in parts of the United States,^133^^,^^134^ and it has a high WNV transmission potential in the laboratory^135^ though field-caught specimens in Ontario rarely test positive for WNV.^24^

#### Aedes togoi.

This review includes the coastal rock pool mosquito, *Aedes* (*Tanakaius*) *togoi* (Theobald), but it should be noted that there is uncertainty about whether *Ae. togoi* is native to North America or is an established invasive species.^136^^–^^139^

### Life history.

*Aedes togoi* breeds in coastal rock pools just above the high tide line that range from freshwater to hypersaline.^140^ In Asia, it has also been reported to breed in artificial containers^109^ but this behavior is absent in North America.^140^^,^^141^ *Aedes togoi* overwinters in the larval stage, the egg stage, or a combination of both depending on the climate,^142^^,^^143^ with populations in North Vancouver overwintering as a combination of both.^144^ North American populations do not generally fly more than 20 m from the shoreline.^141^ Female *Ae. togoi* will blood-feed on humans,^109^ but in North America they are often found in locations that are difficult to access and have no human settlements nearby, indicating that their primary source of blood meals are likely other species.^140^ Due to the reluctance of *Ae. togoi* adults to leave shoreline habitat,^141^ a propensity of *Ae. togoi* larvae to submerge at the slightest provocation and remain hidden in detritus at the bottom of pools for extended periods of time,^140^ and the nature of its habitat leading to difficulties in access, the detection of *Ae. togoi* can prove difficult.

### Distribution.

**Figure 7.**
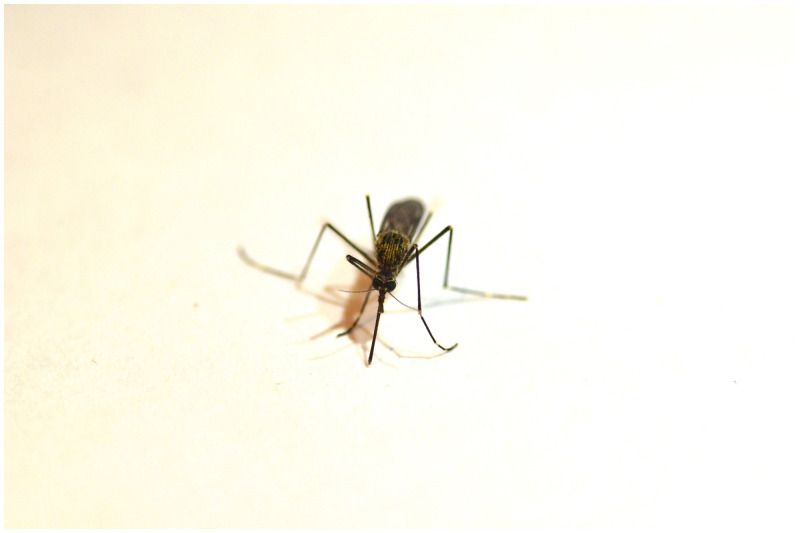
Female *Aedes togoi*, photo by Dan Peach. This figure appears in color at www.ajtmh.org.

*Aedes togoi* is distributed along the coast of east Asia from Malaysia to the Russian Far East in environments that range from subtropics to subarctic.^109^ It is also found in Pacific Canada along the coast of southern British Columbia (Figure 7), and down into Washington, though the northern extent of its range in North America is unknown.^112^^,^^137^^,^^138^ *Aedes togoi* was first detected in North America from Victoria, British Columbia, in 1970,^139^^,^^145^ though atypical records from coastal rock pools as early as 1919 may indicate *Ae. togoi* was observed much earlier but mistaken for other species.^138^^,^^146^ Genetic evidence indicates that populations of *Ae. togoi* in British Columbia belong to a haplotype not found in known populations from Japan, China, or southeast Asia,^136^ implying that they may have originated from populations in Primorsky Krai, Russia, or unknown populations elsewhere in the North Pacific.

### Taxonomy and identification.

**Figure 8.**
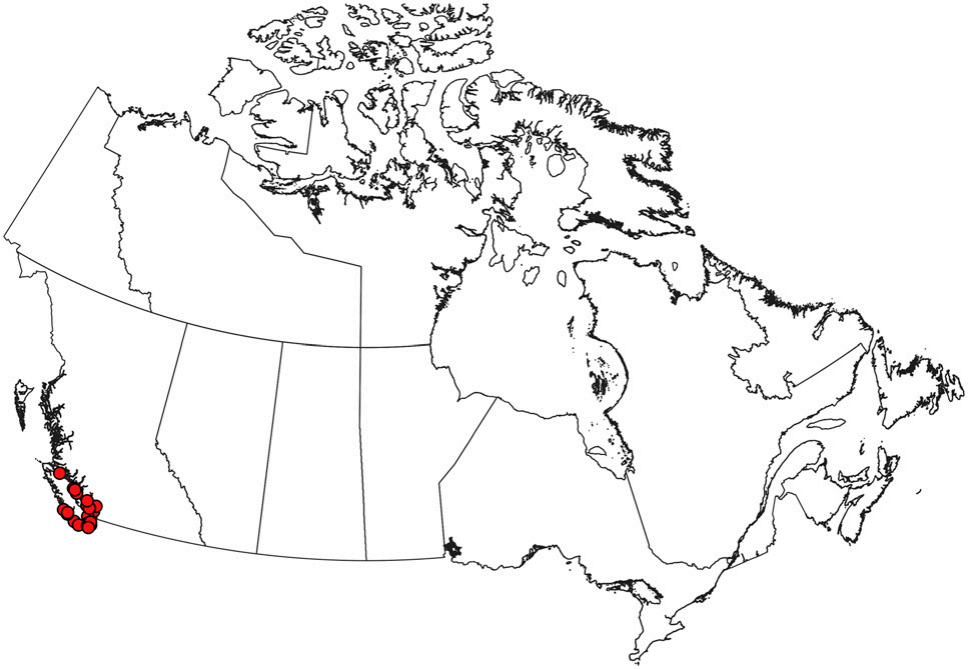
Collection records of *Aedes togoi* in Canada.^112^^,^^139^ This species is distributed along the south coast of British Columbia. The lack of records of *Ae. togoi* from the north coast of British Columbia may represent an information deficiency rather than true absence. This figure appears in color at www.ajtmh.org.

Another dark mosquito with banded legs, *Ae. togoi* has lines of gold scales on the scutum (Figure 8) and is one of the few mosquitoes commonly found in its extreme coastal habitat. *Aedes japonicus* can be mistaken for *Ae. togoi* due to similar patterns of scales on the legs and scutum, but the bands of pale scales on the tarsomeres of *Ae. togoi* are present at the base and apex of each tarsomere, whereas those of *Ae. japonicus* are present only at the base of each tarsomere.^2^^,^^82^

### Public health and veterinary importance.

In Asia, *Ae. togoi* is a vector of the filarial parasite *Brugia malayi*,^109^^,^^147^^,^^148^ JEV,^53^^,^^149^^,^^150^ and potentially the parasites *Wuchereria bancrofti* and *Dirofilaria immitis*.^109^ However, except in some specific locations, it seems to be secondary in importance to other vector species.

### *Culex pipiens* life history.

The northern house mosquito, *Culex pipiens*, can breed in almost any type of standing water, particularly containers such a birdbaths, gutters, or buckets, as well as nutrient-rich, stagnant, or polluted water such as ditches or even sewage ponds.^4^^,^^151^^,^^152^ Eggs are deposited as a floating raft on the water’s surface, and larvae can develop into adults in 1–2 weeks.^4^^,^^151^ Adult *Cx. pipiens* are somewhat variable in their hours of peak biting activity but are generally crepuscular to nocturnal.^153^ This species is primarily ornithophilic, though they do occasionally bite mammals, including humans.^20^

*Culex pipiens* numbers build up over the summer, reaching their peak in late July and early August before tapering off into the fall.^116^ They feed on nectar from a variety of flowers^154^ and can pollinate some members of the Asteraceae.^155^ Overwintering occurs as sugar-fed, inseminated nonblood-fed females, which enter warm locations such as human buildings or urban storm water drains in the fall to shelter until they emerge in the spring to feed.^156^^,^^157^

### Distribution.

**Figure 9.**
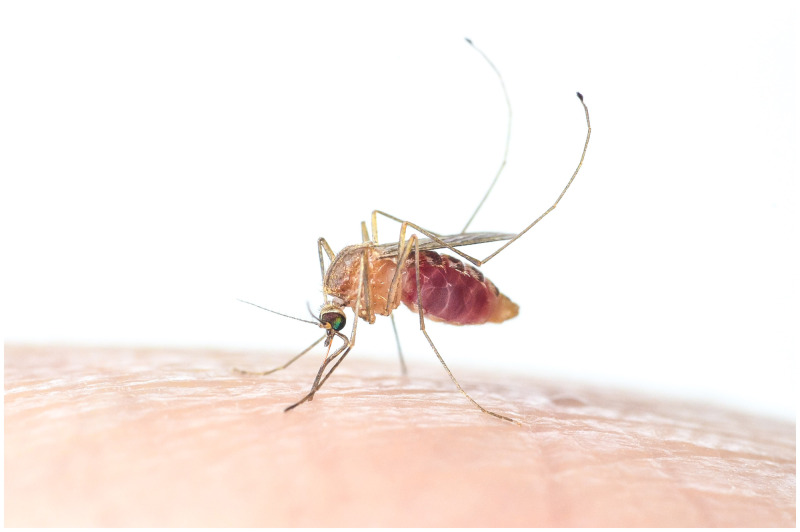
Female *Culex pipiens*, photo by Adam Blake. This figure appears in color at www.ajtmh.org.

*Culex pipiens* is a widespread invasive species that has spread throughout the Holarctic as well as into Australia, parts of South America, and South Africa.^4^^,^^151^ In Canada, this species is known from Nova Scotia, New Brunswick, Prince Edward Island, Southern Quebec, Southern Ontario, and British Columbia^4^^,^^82^^,^^114^^,^^158^^,^^159^ (Figure 9). It has been reported from prairie provinces but these records are not supported by specimens or subsequent collections.^4^^,^^160^ Present in North America for centuries, *Cx. pipiens* perhaps arrived in eastern North America as early as the late fifteenth century.^161^ However, this species is believed to have spread to Newfoundland only in the early twenty-first century^159^ and is thought to have been introduced into British Columbia in the early twentieth century, as it was not found in 1904^162^ and in 1926. *Culex pipiens* was found only at one location in British Columbia.^163^ Perhaps, as a result of its use of heated human structures for overwintering, this species is established as far north as Prince George, British Columbia,^164^ making it the invasive mosquito with the most northernly known distribution in North America. This species had also been reported at Sitka and Yakutat, Alaska, as *Cx. consobrinus* in the summer of 1899,^165^ though subsequent Alaskan records have not been reported.^82^

Over the coming decades, environmental conditions are predicted to become suitable for *Cx. pipiens* in parts of Alberta, Saskatchewan, and Manitoba, as well as Western Ontario.^160^ Due to its ability to use human structures to escape winter conditions, as well as predictions that future climate conditions will be more favorable to this species in many parts of Canada,^160^ this species is likely to spread further within Canada.

### Taxonomy and identification.

**Figure 10.**
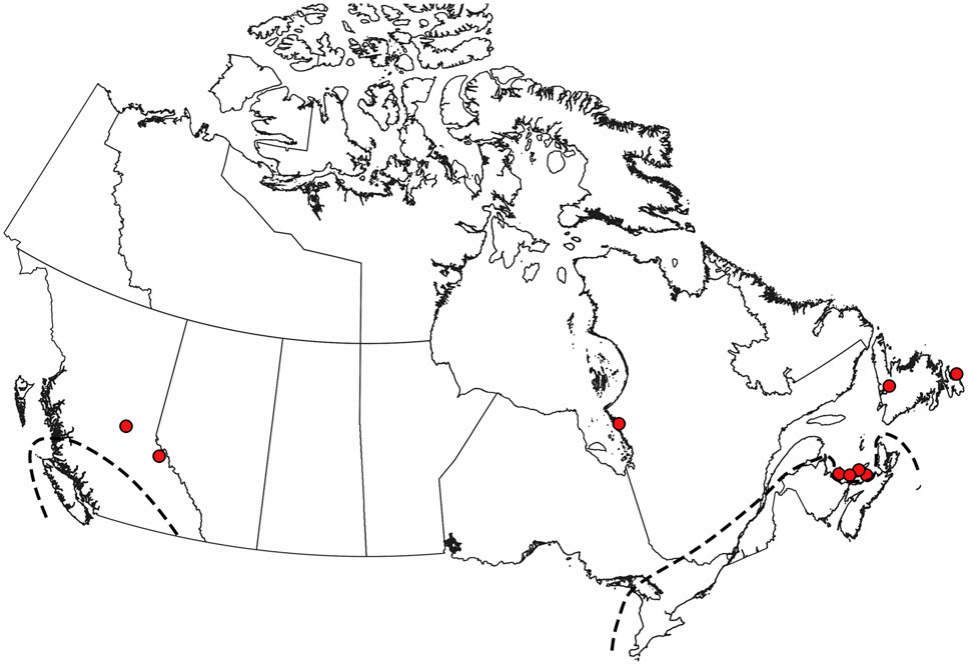
Distribution of *Culex pipiens* in Canada including the recognized distribution limits of *Cx. pipiens* in Canada as of 2005^82^ (hash marks), with recent additional collection records.^158^^,^^159^^,^^164^^,^^219^ The record from near Chisasibi, QC, may not represent an established population and requires further investigation. This figure appears in color at www.ajtmh.org.

A small brown mosquito without distinct markings (Figure 10), adult female *Cx. pipiens* can be difficult to reliably distinguish from similar species such as *Cx. restuans* without molecular tools. However, if specimens are in pristine condition, the presence of patches of pale scales on the scutum can distinguish *Cx. restuans* from *Cx. pipiens*.^2^^,^^4^^,^^82^ The amphibian biting *Cx. territans*, another species that appears similar, possesses bands of pale scales on the apices of dorsal abdominal segments, whereas these bands are basal in *Cx. pipiens*.^2^^,^^4^^,^^82^^,^^137^ A sister species of *Cx. pipiens*, the southern house mosquito, *Cx. quinquefasciatus*, must be separated from *Cx. pipiens* by molecular techniques or examination of male genitalia; however, *Cx. quinquefasciatus* is not known from Canada.^82^

### Public health and veterinary importance.

Though not an aggressive biter,^166^ *Cx. pipiens* is an important vector of WNV and is considered as the primary bridge vector of WNV between birds and humans in northeastern North America.^20^^,^^21^^,^^23^^,^^24^ It is also a vector of SLEV,^167^^,^^168^ USUV,^169^ avian malaria,^170^^–^^172^ and dog heartworm.^173^ Similar to human malaria, avian malaria can have massive ecological impacts on the organisms it affects. Declines in populations of the house sparrow, *Passer domesticus*, in many areas of Europe are thought to be due to *Plasmodium relictum*^174^ and the introduction of this parasite into the Hawaiian Islands has devastated endemic bird species, with some experiencing disease mortality of 90% and at least one extinction connected to *P. relictum*.^175^^–^^178^ The ecology of avian malaria transmission and its impacts need more investigation by vector ecologists, particularly in the northern area of its range and along bird-migration routes.

### Future considerations.

#### Invasive species to watch for.

*Aedes atropalpus* is native to Eastern North America where it was originally limited to breeding in rock pools.^4^ Since the late 1970s, it has begun using discarded tires as breeding habitat^179^ and has become invasive in parts of the American Midwest^179^ as well as Europe.^180^^,^^181^ By breeding in discarded tires is possible that *Ae. atropalpus* could invade parts of Canada it has not historically inhabited.

*Aedes koreicus* is closely related to *Ae. japonicus* and is abundant in urban settings.^182^ The native range of *Ae. koreicus* is in eastern Asia, including China, Korea, and eastern Russia,^109^ but it has become established in Europe where it is believed to have arrived in used tires.^128^^,^^183^ As a container-breeding species well-adapted to temperate climates, human-dominated environments, and a history of spreading to new areas, *Ae. koreicus* may have the potential to invade and establish in Canada.

*Culex tritaeniorhynchus* is native to Asia and parts of Africa^109^ and is the primary vector of JEV virus in many parts of Asia.^109^ This species has been found on ships as far as several hundred kilometers out to sea^184^ and, while it primarily breeds in other habitats, its larvae have occasionally been found in containers.^185^ *Culex tritaeniorhynchus* could arrive as ship-borne adults or as larvae in containers shipped from its native habitat.

## DISCUSSION

With an increasingly interconnected world, the establishment of invasive alien species is on the rise worldwide.^186^ At least half of the invasive mosquitoes known from Canada have arrived within the last 20 years, and all but one since the middle of the twentieth century. Mosquito and pathogen monitoring and control in Canada must adapt to these challenges by utilizing modern solutions and undertaking context-specific research.

As genomic resources for mosquitoes continue to improve, the ability to identify species via molecular means will become increasingly important in surveillance and control efforts. Widely deployed molecular identification (e.g., through “DNA barcoding”^187^) is a critical component of vector surveillance, as it can resolve cryptic or sibling species as well as reduce the need for highly trained observers. Furthermore, molecular identification can be performed on partial or damaged samples from multiple life stages,^188^ obviating the need for pristine specimens for morphological identification. Pathogen surveillance programs have traditionally relied upon morphological identification techniques, which have resulted in the successful detection of several previously unknown native mosquitoes^3^^,^^189^ and novel invasives.^5^^,^^113^ However, DNA barcoding methods were recently used in a province-wide survey of mosquitoes in Quebec^118^ and we propose that similar methods should be routinely and widely implemented (in tandem with morphological identification techniques) as part of mosquito surveillance efforts across Canada. In addition to species identification, sequencing analysis can help to identify the likely source of invasive mosquitoes,^63^^,^^64^^,^^190^^,^^191^ which will aid in targeting surveillance and trapping to detect and prevent further incursion (e.g., at ports or airports). Finally, sequencing intact mosquito specimens can identify arbovirus infection status and bloodmeal host identity^73^^,^^192^^–^^198^ providing critical information to aid in control efforts. We propose that the generation of high-quality reference genomes should be prioritized for mosquitoes native and invasive to Canada. Contiguous and complete genome assemblies can now be readily constructed from as little as a single mosquito due to improvements in long-read sequencing technologies and library preparation protocols.^199^^–^^201^ The availability of comprehensive and complete genomic resources will help to better understand the biology of these species and to guide the development of molecular assays for determining insecticide resistance status.^202^^–^^205^ Further, coupled with whole-genome sequencing of additional specimens, a complete genome assembly is a foundation identifying the genomic basis of oviposition behavior, blood-meal host preference, and other phenotypes that may influence the ability of specific populations to take hold and adapt to new niches.^206^ However, it is important to stress that molecular techniques do not obviate the importance of morphological techniques in vector surveillance and at broader scales,^207^^,^^208^ and that future best practices will involve a reciprocal and integrative interaction between these methodologies.^209^

Broad collaboration and data-sharing are imperative to monitor, prevent, and prepare for the spread of invasive mosquitoes and the pathogens they vector.^210^^–^^212^ Requirements for open data policies combined with a greater resolution of data, such as species-level identifications, for such institutions that monitor for mosquitoes and pathogens, and their contractors, are one mechanism by which to address these needs. Detailed data collection and open data policies regarding species and pathogen presence will also inform modeling efforts and risk assessments, policy decisions, and healthcare initiatives. To facilitate these efforts, and build upon pathogen monitoring activities such as those carried out by provincial CDC, the Public Health Agency of Canada, Public Health Ontario, and others, we propose the formation of a Canada-wide network for sharing information on the distribution of mosquito vectors and the pathogens they spread, similar to the VectorNet (https://vectornet.ecdc.europa.eu/) and VBORNET (www.vbornet.eu) initiatives established by the European Center for Disease Prevention and Control and European Food Safety Authority. Additionally, monitoring for invasive mosquitoes arriving at seaports, airports, and other areas at which high volumes of international traffic arrive, as has been done in other nations^213^^–^^216^ and in some limited parts of Canada such as southern Ontario,^5^^,^^26^ should be considered to enhance Canada’s biosecurity due to the ease at which some invasive mosquitoes spread within a country once established.^91^^,^^110^^,^^217^^,^^218^

Furthermore, Canada provides a unique opportunity to study invasive mosquitoes and their pathogens under an environment that is changing in both climate and levels of human modification. Many of these mosquitoes and pathogens are at the northern limits of their range in Canada and are likely experiencing immense selection pressures on behavioral and physiological mechanisms to survive cold winters. With changing climatic conditions and increasing global connectivity, there are also unique opportunities to study how invasive mosquitoes and exotic pathogens arrive and spread in new areas, as well as the effect of urbanization and temperature limitations on mosquito and pathogen community composition, mosquito behavior, and pathogen transmission dynamics.
